# Touchscreen-based location discrimination and paired associate learning tasks detect cognitive impairment at an early stage in an *App* knock-in mouse model of Alzheimer’s disease

**DOI:** 10.1186/s13041-020-00690-6

**Published:** 2020-11-13

**Authors:** Md. Ali Bin Saifullah, Okiru Komine, Yutao Dong, Kazuya Fukumoto, Akira Sobue, Fumito Endo, Takashi Saito, Takaomi C. Saido, Koji Yamanaka, Hiroyuki Mizoguchi

**Affiliations:** 1 Research Center for Next-Generation Drug Development, Research Institute of Environmental Medicine, Nagoya University, Nagoya, Aichi, 464-8601 Japan; 2 Department of Neuroscience and Pathobiology, Research Institute of Environmental Medicine, Nagoya University, Nagoya, Aichi, 464-8601 Japan; 3 Department of Neuropsychopharmacology and Hospital Pharmacy, Nagoya University Graduate School of Medicine, Nagoya, Aichi, 466-8560 Japan; 4 Laboratory for Proteolytic Neuroscience, RIKEN Center for Brain Science, Wako, Saitama 351-0198 Japan; 5 Department of Neurocognitive Science, Institute of Brain Science, Nagoya City University Graduate School of Medical Sciences, Nagoya, Aichi 467-8601 Japan

**Keywords:** Alzheimer's disease, Touchscreen, Amyloid beta, Early stage, *App*-KI

## Abstract

Alzheimer's disease (AD) is a progressive neurodegenerative disorder characterized by cognitive decline with accumulation of amyloid beta (Aβ) and neurofibrillary tangles that usually begins 15–30 years before clinical diagnosis. Rodent models that recapitulate aggressive Aβ and/or the pathology of neurofibrillary tangles are essential for AD research. Accordingly, non-invasive early detection systems in these animal models are required to evaluate the phenotypic changes, elucidate the mechanism of disease progression, and facilitate development of novel therapeutic approaches. Although many behavioral tests efficiently reveal cognitive impairments at the later stage of the disease in AD models, it has been challenging to detect such impairments at the early stage. To address this issue, we subjected 4–6-month-old male *App*^*NL*−*G*−*F/NL*−*G*−*F*^ knock-in (*App*-KI) mice to touchscreen-based location discrimination (LD), different object–location paired-associate learning (dPAL), and reversal learning tests, and compared the results with those of the classical Morris water maze test. These tests are mainly dependent on the brain regions prone to Aβ accumulation at the earliest stages of the disease. At 4–6 months, considered to represent the early stage of disease when mice exhibit initial deposition of Aβ and slight gliosis, the classical Morris water maze test revealed no difference between groups, whereas touchscreen-based LD and dPAL tasks revealed significant impairments in task performance. Our report is the first to confirm that a systematic touchscreen-based behavioral test battery can sensitively detect the early stage of cognitive decline in an AD-linked *App*-KI mouse model. This system could be applied in future translational research.

## Introduction

The advancement of modern medical science has increased life expectancy and led to an increase in the aging population. As a result, age-related diseases are becoming much more common. One of the most common age-related neurodegenerative disorders is Alzheimer’s disease (AD). This debilitating disease is mainly caused by accumulation of extracellular Aβ and intracellular neurofibrillary tau tangles. Emerging data suggest that the disease process begins years before clinical diagnosis. The disease has a long preclinical phase with no clinical symptoms, followed by an early phase, also known as mild cognitive impairment phase, associated with mild symptoms, and a disease phase, when cognitive impairment becomes evident [1–3]. Early detection of the disease is important for effective intervention, including counseling, cognitive training, and medication [4]. Clinical studies have shown that the benefit of currently available medications is higher when initiated in the early phase of the disease [5, 6].

Basic AD research relies largely on various transgenic mouse models that experience accelerated accumulation of Aβ and tau tangles. We have also demonstrated the mechanism of cognitive impairment in an animal model of AD and provided an effective approach for treatment of AD [7–10]; however, these mice exhibit artificial phenotypes and pathologies that are not present in human AD [11, 12]. To overcome these undesired phenotypes, we decided to utilize *App*^*NL−G−F/NL−G−F*^ (*App-*KI) mice, a new AD mouse model that overproduces Aβ_42_ without overexpressing amyloid precursor protein (APP) [13]. *App*^*NL−G−F*^ mice were generated by introducing three familial AD-associated mutations at the endogenous mouse *App* locus by the knock-in approach [13]. *App*-KI mice exhibit aggressive Aβ plaque deposition in the cortical and hippocampal regions at the age of 4 months.

Non-invasive cognitive tests are useful tools for identifying subjects with a high risk of developing symptomatic AD. Although *App-*KI mice have been subjected to various classical behavioral tasks to evaluate various cognitive parameters, detection of cognitive impairment at the earliest stage has been a challenge [14–17]. Moreover, the majority of these tests have low translational value because the testing conditions differ from those used for patients with AD. Recently, touchscreen-based behavioral testing systems have been developed that employ similar stimuli (images displayed on the screen) and reactions (touch) to assess performance, thereby ensuring an analogous testing system for humans and other disease model [18]. Hence, in this study we sought to detect cognitive impairments of *App-*KI mice associated with the early stage of AD in touchscreen-based behavioral tasks. We selected behavioral tests that require cortical and/or hippocampal function, as these regions are affected at the earliest stage in *App-*KI mice. Our results revealed that hippocampus-dependent touchscreen-based behavioral tasks could detect AD at an early stage for the first time, and also confirm the potential use of these cognitive tests in developing therapeutic approaches for preventing AD progression.

## Materials and methods

### Animals

Generation of *App*-KI (*App*^*NL*−*G*−*F/NL*−*G*−*F*^) mice was described previously [13]. Mice were obtained from the RIKEN Center for Brain Science (Wako, Japan). Male *App*^*NL*−*G*−*F/NL*−*G*−*F*^, littermate, and wild-type (C57BL/6 J, CLEA Japan, Inc., Japan, RRID:IMSR_JAX:000,664) mice were 4 months old at the start of touchscreen experiments and 6 months old for the Morris water maze test. All mice were housed in plastic cages and kept in a regulated environment (23 ± 1 °C; 50 ± 5% humidity) with a 12-h light/dark cycle (lights on at 8:00 AM). Food (CE-2; CLEA Japan, Inc.) and tap water were available ad libitum. All experiments were performed in accordance with the Guidelines for Animal Experiments of Nagoya University, the Guiding Principles for the Care and Use of Laboratory Animals approved by the Japanese Pharmacological Society and the United States National Institutes of Health Guide for the Care and Use of Laboratory Animals. All experimental procedures were approved by the Institutional Animal Care and Use Committee of the Research Institute of Environmental Medicine, Nagoya University (Permit Number: RIEM19273). One week before starting behavioral experiments, mice were food-restricted to achieve approximately 85% of their ad libitum bodyweight. Mice that exhibited severe bodily injury due to fighting were excluded from behavioral analysis.

### Touchscreen apparatus

Touchscreen tests were performed as previously described with slight modifications [19–21]. Testing was conducted with a touchscreen-based automated operant system for mice housed within a sound- and light-attenuating box (87 × 50 × 79 cm, TOP-M1, O’hara & Co., Ltd., Tokyo, Japan). The sound-attenuating box contained a house light, a ventilation fan that also provided white noise, and a pair of tone generators. The operant system contained a 15-inch touch panel unit and a 10-mg food pellet dispenser on the opposite side, fitted with a photocell head entry detector and a camera directly on top of the chamber. To decrease the frequency of unintended responses to the touchscreen due to contact with the tail or other body parts, a black plastic “mask” with task-specific response windows was placed in front of the screen: six windows for LD, two windows for visual discrimination and reversal learning, and three windows for different object–location paired-associate learning (dPAL) touchscreen-based behavioral tasks.

### Pretraining

Before performing any of the touchscreen tests described in this study, mice had to go through pre-training steps, which consisted of the following: (1) Magazine training (1 day, 30 min), in which mice received food (10-mg pellet, AIN-76A Rodent Tablet 10 mg, #1811213 (5TUL), TestDiet, USA) for head entry into the food receptacle. (2) Autoshaping (1 day, 30 min), in which food was delivered after contingent disappearance of presentation of a white stimulus in all windows. (3) Must touch (2 days, 60 min/100 trials), in which mice had to touch the screen to receive a food reward; all windows presented white stimuli; (4) Correct touch (2 days, 60 min/100 trials), in which mice had to touch the stimulus presented randomly in only one window to receive a food pellet; incorrect response had no effect. (5) Correct touch error (2 days, 60 min/100 trials, at least 80% correct response), in which mice had to correctly touch the randomly presented white stimulus to receive a food reward. Pretraining required 10 sessions and data from pretraining sessions are not shown.

In all pretraining steps and touchscreen tests, the trial was automatically started followed by a 3-s inter-trial interval (ITI), after which the mouse was required to enter its head into the food receptacle to start the trial, as described previously [21]. Head entry into the magazine during a session resulted in stimuli being displayed. A stimulus remained on the screen until the mouse responded to it, after which (if the choice was correct) the mouse was rewarded with a pellet accompanied by a tone, the magazine light was illuminated, and the trial was ended. This was followed by a 3-s ITI before starting the next trial. The house light was on during the trial. After a choice was made, the first head entry into the magazine after the ITI resulted in the stimuli being displayed for the next trial. This meant that on every trial, the mouse was situated at the back of the testing chamber when the stimuli were displayed.

### Location discrimination (LD) task

**Fig. 1:**
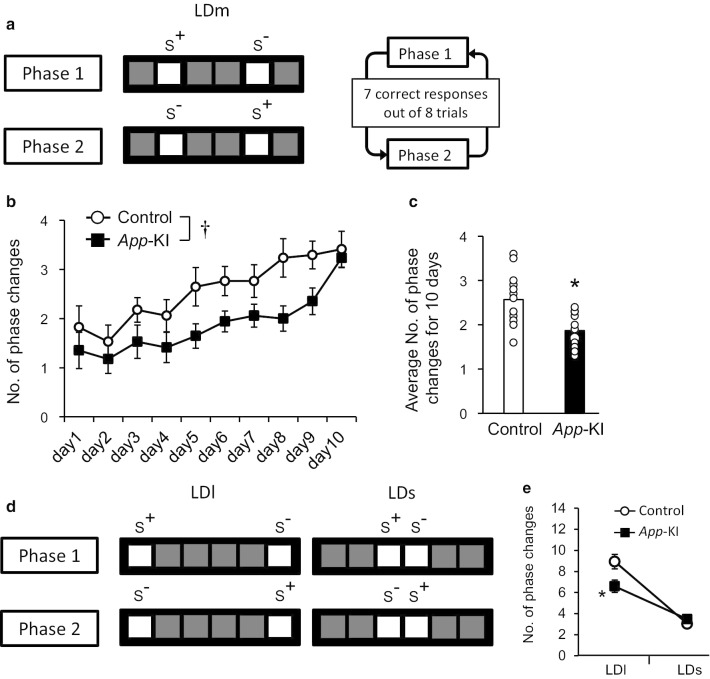
Impaired pattern separation of *App*-KI mice in the LD task. **a** Diagram represents location discrimination tasks with a medium degree of separation between stimuli, as well as criteria for phase change. **b**, **c**, Number of phase changes in LDm training sessions in *App*-KI mice (n = 17). Mice were given a maximum of 62 trials/session/d for 10 days. Data are presented as means ± SEM. *, † p < 0.05 vs control. **d** Diagram represents location discrimination tasks with high and low degree of separation between stimuli, as well as criteria for phase change. **e** Number of phase changes during 30-min training sessions in *App*-KI mice (n = 17). Data are presented as means ± SEM. *p < 0.05 vs LDl in control

The LD task was performed as described previously, with slight modification [22]. We used mask and stimulus dimensions as follows: number of windows, 6; window size, 25 × 25 mm^2^, window gaps, 10 mm; floor gap, 25 mm; stimulus size, 25 × 25 mm^2^. Following pretraining sessions, the LD task included an additional training step in which the mice were presented with two square white stimuli separated by an intermediate degree of separation (LDmedium: LDm). One square was designated as correct, and the other as incorrect (Phase 1). Responses at the correct location resulted in a reward delivery followed by 3-s ITI as described above. Responses at the incorrect location resulted in a 5-s timeout period with the house light off. Seven correct responses out of eight consecutive trials resulted in reversal of reward contingences, in which the previous incorrect location now became correct (Phase 2) (Fig. 1a). The initial correct location was counter balanced between animals in each genotype. Mice were given a maximum of 62 trials/session/d for 10 days. Following LDm training, pattern separation was assessed by presenting stimuli with either an LDlarge (LDl) task with a high degree of separation (four empty windows between two stimuli) or an LDsmall (LDs) task with a low degree of separation (no empty windows between the two stimuli). Mice received a 30-min once daily session. Mice were subjected to each task for 4 days. The same degree of separation was presented for two consecutive days. The order of separation was counterbalanced between animals in each group across days. The average number of changes between phases during the LDl and LDs tasks were calculated. The LD task required 20 training sessions: 10 sessions for the LDm task and 10 sessions for the LDs and LDl tasks.

### Different object–location paired-associate learning (dPAL) task

**Fig. 2:**
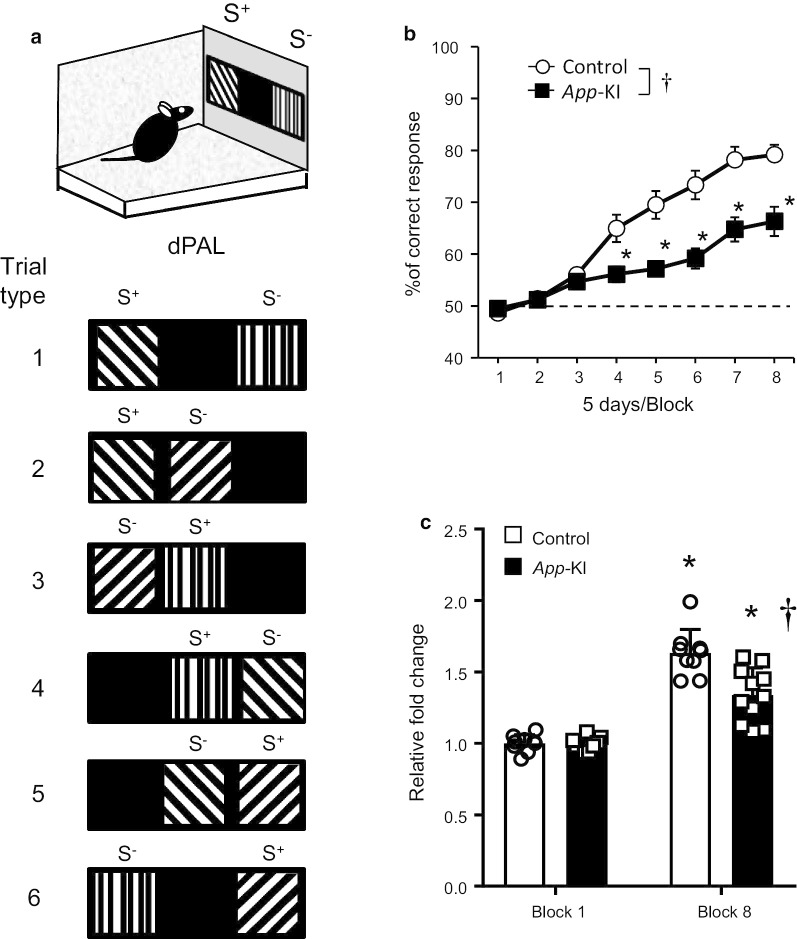
Impaired associative memory of *App*-KI mice in the dPAL task. **a** Diagram represents all possible combinations of stimuli presentation. **b** Five-day blocks of performance during acquisition of the dPAL task. *App* -KI mice exhibited significantly worse performance than wild-type mice. †p < 0.05 vs control. *p < 0.05 vs each block in control. **c** Fold change relative to the first block. A significant difference was observed in block 8, although both groups of mice exhibited significant improvement in the last data point relative to the first data point. †p < 0.05 vs control in block 8. *p < 0.05 vs block 1 in each group. Data are presented as means ± SEM (n = 9–11). Chance performance (50%) is indicated by dashed lines

We used mask and stimulus dimensions as follows: number of windows, 3; window size, 57 × 57 mm^2^; window gaps, 10 mm; floor gap, 25 mm; stimulus size, 53 × 53 mm^2^. Following pretraining sessions, dPAL tasks also had one additional training step in which place-associated stimuli, were presented but the incorrect response had no effect. The dPAL experiment was performed as previously described [23]. Briefly, six different combinations were designed using three lined stimuli. Each stimulus was considered S^+^ in a specific location. For each trial type, one visual stimulus was presented in its correct location and a second visual stimulus was presented in an incorrect location, leaving one window blank (Fig. 2a). Each combination of stimuli was presented an equal number of times. A correct choice was followed by reward delivery with tone, illumination of the magazine light, a 3-s ITI, and the next trial. An incorrect choice was followed by a 5-s timeout and a 3-s ITI, after which correction trials started in which the same set of stimuli are presented until the correct choice was made. Correction trials were not included in the calculation of percent correct response. The session finished either when the 120 trials were completed or when 60 min had passed. The dPAL task required 40 training sessions.

### Visual discrimination, and reversal learning task

**Fig. 3:**
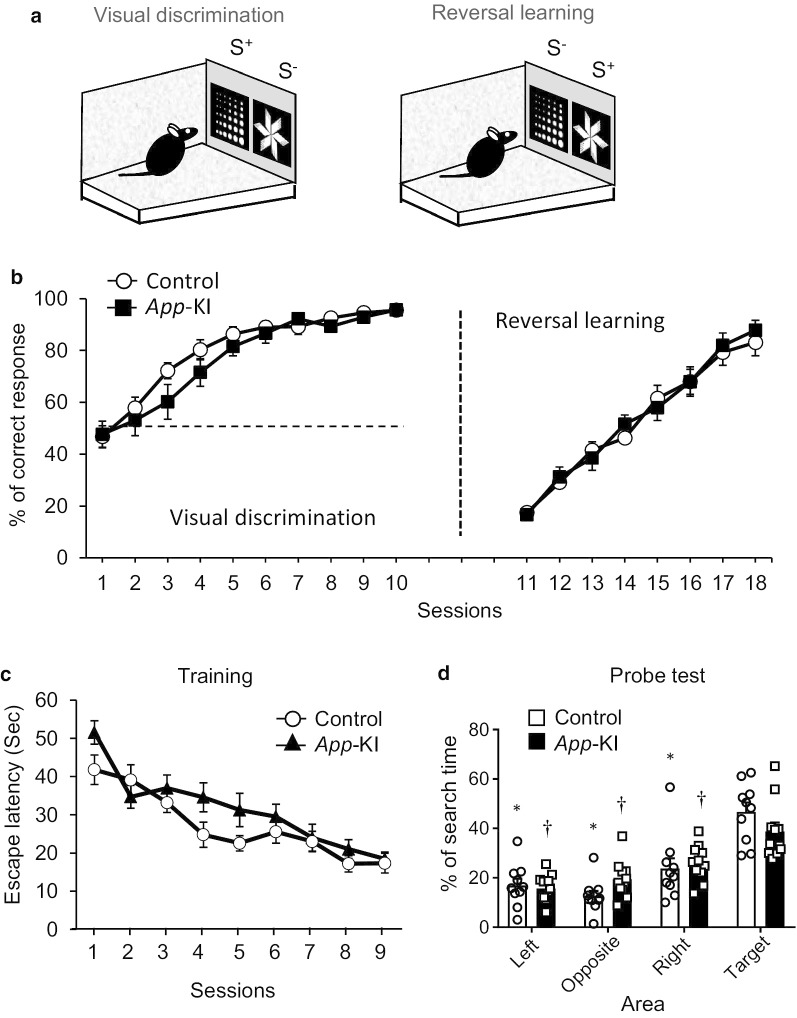
*App*-KI mice exhibit no changes in cognitive flexibility or reference memory. **a**, **b** Visual discrimination, reversal learning in the touchscreen-based operant system. **a** Diagram representing visual discrimination and reversal learning. **b** Behavioral performance of 4–5-month-old mice in both tasks. Data are presented as means ± SEM (n = 10–11). Chance performance (50%) is indicated by dashed lines. **c**, **d** Reference memory in Morris water maze test. **c** In 6-month-old mice, escape latency was measured during a 60-s session in the water maze test. **d** Spatial memory of a platform’s location during the training phase was tested in the probe test. Percentage of time spent in each quadrant was measured. Data are presented as means ± SEM (n = 10–11). *p < 0.05 vs target in WT mice (Tukey test for multigroup comparisons). †p < 0.05 vs target in *App*-KI mice (Tukey test for multigroup comparisons)

*Acquisition* Visual discrimination and reversal learning tests were performed as described previously [24] with slight modifications. We used mask and stimulus dimensions as follows: number of windows, 2; window size, 60 × 60 mm^2^; window gap, 30 mm; floor gap, 25 mm; stimulus size, 60 × 60 mm^2^. Mice were presented with a pair of black-and-white, brightness-matched stimuli on the touchscreen, one of which was correct (S^+^) and the other incorrect (S^−^) (Fig. 3a). Response to the S^+^ resulted in a tone, magazine illumination, and delivery of a single reward pellet. After incorrect responses, the house light was extinguished. Both correct and incorrect responses were followed by a 3-s ITI. Each daily session consisted of 100 trials in 60 min. Percent correct response per 100-trial session was calculated to evaluate performance. In this stage, although the animal reached an 80% correct response rate by the 5^th^ training session, we continued training to ensure that both groups of animals reached the highest level of learning.

*Reversal* After reaching the acquisition criteria, in following sessions, reward contingencies of S^+^ and S^−^ were reversed (Fig. 3a). In this phase, the previously unrewarded stimulus provided reward. Each training session had parameters similar to those in acquisition phase. The reversal phase continued until one or both groups reached ~ 80% correct responses for 2 consecutive days, which required 8 sessions.

### Morris water maze test

The Morris water maze test was performed as previously described [8] with minor modifications. Briefly, a circular pool 1.2 m in diameter was filled with water at a temperature of 22 ± 1 °C. A transparent platform (7 cm in diameter) was submerged inside the pool. Objects of different shapes were placed on the surrounding walls. The mice were trained in three 60 s sessions for 9 days, during which the platform and the objects on the walls were fixed in the same position. Twenty-four hours after the last training trial, the mice were given a probe test without the platform and were allowed to search the platform for 60 s. Mice that did not swim were excluded from all behavioral experiments. The time taken to locate the escape platform (escape latency) and the distance moved was determined in each trial using the SMART system (SMARTBASIC / SMART 3.0 BASIC PACK, Panlab, Barcelona, Spain). Nine training sessions were required to reach stable performance. Mice that exhibited odd behaviors such as spinning, lack of swimming, staying close to the periphery, or being unable to find the platform before time ran out after 6 days of training in the Morris water maze test were excluded from all behavioral experiments.

### Immunohistochemistry

Mice were deeply anesthetized by high-concentration isoflurane for animal (MSD Animal Health K.K., Tokyo, Japan) and perfused with 4% paraformaldehyde in phosphate buffer (4% PFA). Brains were dissected and post-fixed with 4% PFA for 24 h, and then cryoprotected in 30% sucrose in PBS for 24 h. Twelve-micron sagittal cryosections were prepared and treated with HistoVT One (Nacalai Tesque, Japan) at 70 °C for 20 min. The sections were then pre-incubated with 5% normal donkey serum/0.3% Triton-X-100 in PBS for 1 h and immunostained with primary antibodies against Iba-1 (1:250, Novus Biologicals, USA, RRID:AB_521594), GFAP (1:500, DAKO, Denmark, RRID:AB_10013382), and the N-terminal region of human Aβ conjugated with biotin (1:200, IBL, Japan, RRID:AB_10705565) followed by Alexa Fluor 488–conjugated anti-goat IgGs (1:1000, Invitrogen, USA, RRID:AB_2534102), Alexa Fluor 647–conjugated anti-rabbit IgGs (1:500, Jackson ImmunoResearch Laboratories, USA, RRID:AB_2492288), and Alexa Fluor 546–conjugated streptavidin (1:500, Invitrogen, USA, RRID:AB_2532130). Images were obtained on a confocal laser microscope (LSM700, Carl-Zeiss, Germany).

Immunostaining for analysis of hippocampal neurogenesis was performed as described previously with slight modifications [25]. Twenty-five–micron coronal frozen sections were post fixed with 4% PFA for 20 min and washed three times with PBS. They were incubated with methanol for 30 min, and then with 0.3% Triton-X/PBS buffer for 30 min at 37 °C. They were then autoclaved with Antigen Unmasking Solution (H-3300, Sigma-Aldrich, RRID:AB_2336226) at 105 °C for 2 min, followed by three washes with PBS. They were incubated for 30 min in blocking serum (10% normal goat serum in 0.3% Triton-X 100/PBS) and then for 24 h at 4 °C in the presence of a primary antibody against doublecortin (DCX) (E-6) (a neuronal lineage marker) (1:100, sc-271390, Santa Cruz Biotechnology, Dallas, TX, USA, RRID:AB_10610966) and Ki-67 (SP6) (a proliferating cell marker) (1:100, ab16667, Abcam, Cambridge, MA, USA, RRID:AB_302459). Sections were then washed three times with 0.05% tween in PBS, incubated in a secondary antibody (Alexa 488, RRID:AB_143165 and Alexa 546, RRID:AB_144695) (1:1000, Invitrogen, USA) for 2 h, and washed three times with 0.05% Tween in PBS. Antibodies were diluted in the Can Get Signal® immunostain Solution A (NKB-501, TOYOBO, Japan).

The dentate gyrus (DG) was segregated into dorsal regions (approximately − 1.8 to − 2.3 mm from bregma) [26], and cells in each segregation were quantified to determine any difference in neurogenesis between groups. Samples were observed with a microscope (BZ-9000, KEYENCE Corp., Osaka, Japan) and analyzed at 40 × magnification. The number and density of cells positive for immunoreactivities were analyzed using ImageJ. The values were summed and divided by the number of slices analyzed for each animal. Four areas of interest (362.99 µm × 273.31 µm), two each in the right and left DG, were imaged on one slice, and averages of at least five slices (20 areas) in each mouse were counted within areas of interest and used for statistical analysis.

### Statistical analysis

All data are expressed as means ± SEM. Statistical analyses were performed with GraphPad Prism 7 (GraphPad Software, San Diego, CA, USA, RRID:SCR_002798). Statistical significance (p < 0.05) was determined using Student’s *t*-test for comparisons between two groups; two-way analysis of variance (ANOVA) for multigroup comparisons; or repeated-measures ANOVA. Bonferroni test and Tukey test were used for post hoc comparison when the F value was significant. The sample size for each experiment was determined based on our previous studies using the relevant type of experiment [7, 8, 27].

## Results

### The LD task is a useful method for detecting impairment in pattern separation in *App*-KI mice at the early stage

We subjected 4–5-month-old *App-*KI mice to the LD task. In the LDm training sessions, mice were given a maximum of 62 trials/session/d for 10 days. Statistical analysis revealed a significant difference between the two groups [Fig. 1b, two-way ANOVA with repeated measures, group, F(1, 32) = 20.22, p < 0.0001, day, F(9, 288) = 15.16, p < 0.0001, interaction, F(9, 288) = 1.03, p = 0.4157; Fig. 1c, t(32) = 4.497, p < 0.0001 by *t*-test], indicating that *App*-KI mice exhibited poorer performance than the control group. On day 10, we observed the same level of performance between the two groups in LDm training. Following LDm training, mice performed both LDl and LDs tasks. We observed a significant difference in behavioral performance only in the LDl task, whereas both groups of mice performed similarly in the LDs task [Fig. 1e, two-way ANOVA followed by Tukey test, group, F(1, 64) = 3.305, p = 0.0738, task, F(1, 64) = 75.99, p < 0.0001, interaction, F(1, 64) = 7.791, p = 0.0069].

### The dPAL task is a useful method for detecting impairment in associative memory in *App*-KI mice at the early stage

Because *App-*KI mice exhibited behavioral impairment in the hippocampus-dependent pattern separation in the LD task, we sought to assess their performance in other hippocampus-dependent tasks. Therefore, we next subjected *App-*KI mice to the dPAL task. In this task, animals learn to associate three different stimuli with their correct spatial locations (Fig. 2a). Learning performances from five sessions were combined to yield a single data point. Relative to the control group, *App-*KI mice exhibited significantly impaired learning [Fig. 2b, two-way ANOVA with repeated measure followed by Bonferroni test, group, F(1, 18) = 16.67, p = 0.0007, block, F(7, 126) = 78.00, p < 0.0001, interaction, F(7, 126) = 10.46, p < 0.0001)]. Especially on blocks 4–8, there were significant differences between two groups. Moreover, when fold change relative to the first block was calculated, both groups exhibited significant improvement in block 8 [Fig. 2c, two-way ANOVA with repeated measure followed by Bonferroni test, group, F(1, 18) = 16.37, p = 0.0008, block, F(1, 18) = 109.3, p < 0.0001, interaction, F(1, 18) = 9.981, p = 0.0054].

### Visual discrimination and reversal learning tasks cannot detect cognitive impairment in *App*-KI mice at the early stage

We also subjected 4–5-month-old *App-*KI mice to a visual discrimination and reversal task. During the acquisition phase, animals learned to discriminate between the reward contingent and non-contingent stimuli. In the acquisition phase, general ability to distinguish visual stimuli was assessed. In the reversal phase, cognitive flexibility was required to inhibit the previously learned stimulus–reward association and relearn a new contingency for a familiar stimulus. In both the acquisition and reversal phase, *App*-KI mice performed similarly to the control group [Fig. 3b, visual discrimination: two-way ANOVA with repeated measure, group, F(1, 19) = 0.9875, p = 0.3328, day, F(9, 171) = 76.41, p < 0.0001, interaction, F(9, 171) = 1.322, p = 0.2286; reversal learning: two-way ANOVA with repeated measure, group, F(1, 19) = 0.06718, p = 0.7983, day, F(7, 133) = 93.47, p < 0.0001, interaction, F(7, 133) = 0.4563, p = 0.8644].

### The Morris water maze test cannot detect impairment in spatial reference memory in *App*-KI mice at the early stage

We also evaluated the reference memory of *App-*KI mice at the age of 6 months using the Morris water maze test. At this age, both groups of mice exhibited similar performance in learning the position of the hidden platform [Fig. 3c, two-way ANOVA with repeated measure, group, F(1, 19) = 3.924, p = 0.0623, day, F(8, 152) = 19.53, p < 0.0001, interaction, F(8, 152) = 1.295, p = 0.2501]. Following the training phase, mice were subjected to the probe test, in which the hidden platform was removed. Both groups of mice spent the largest amount of time in the target quadrant where the platform was previously positioned. Although *App-*KI mice spent less time than control mice in the target quadrant, the difference was not significant [Fig. 3d, two-way ANOVA, group, F(1, 76) = 1.233 × 10^–8^, p > 0.9999, area, F(3, 76) = 37.47, p < 0.0001, interaction, F(3, 76) = 2.312, p = 0.0828].

### Aβ plaque deposition, glia accumulation, and impaired adult neurogenesis are much more advanced in *App*-KI mice at 6 months

**Fig. 4:**
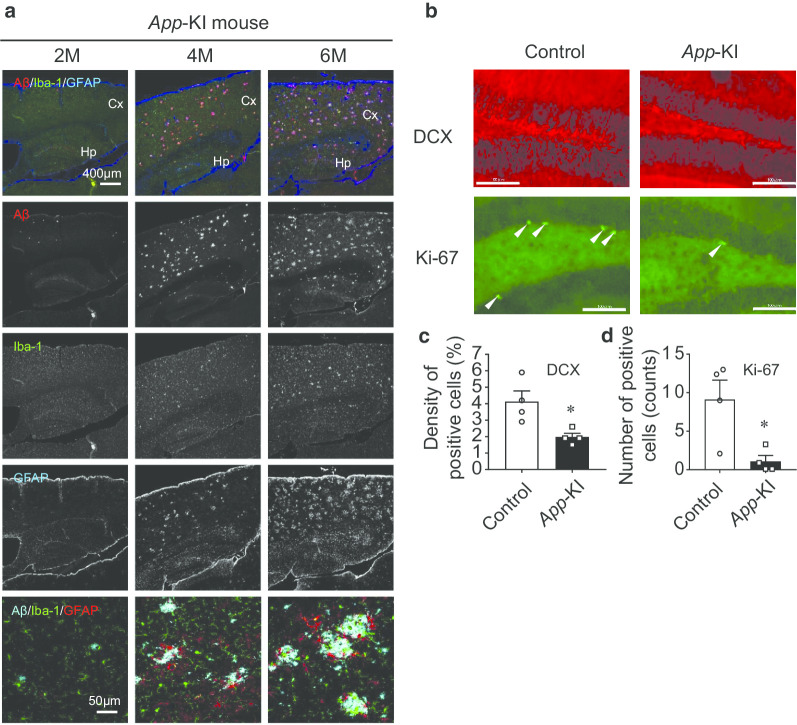
Representative images of amyloid plaque, microglia, and astrocyte expression in *App*-KI mice. **a** Upper panel shows localization of Aβ (red), Iba-1 (green), and GFAP (blue) in the cortico-hippocampus of *App*-KI at 2–6 months age. These are followed by individual images of Aβ, Iba-1, and GFAP (from top to bottom). Bottom panels are representative higher-magnification images of activated microglia and GFAP-reactive astrocytes around Aβ plaques. These panels (Merge) show localization of Aβ (light blue), Iba-1 (green), and GFAP (red) in the cortico-hippocampus of *App*-KI at 2–6 months of age. **b**–**d** Adult neurogenesis in hippocampus of *App*-KI mice. **b** Representative images of DCX and Ki-67 expression. (Scale bar: 100 µm) **c** Quantitative data of DCX-positive cells. Expression levels were reduced in the hippocampus of *App*-KI mice. **d** Quantitation of Ki-67–positive cells. Ki-67-positive cells (arrowheads) were less abundant in hippocampus of *App*-KI mice. Data are presented as means ± SEM (n = 4). *p < 0.05 (*t*-test)

Finally, we examined Aβ plaque deposition in *App*-KI mice at the age of 2, 4, and 6 months. Aβ plaques increased with age in the cortices and hippocampi of *App*-KI mice, and were first observed around 4 months of age. Iba-1- and GFAP-positive cells also accumulated along with Aβ plaques (Fig. 4a). Moreover, we observed the age-dependent presence of activated microglia and GFAP-reactive astrocytes around Aβ plaques (Fig. 4a).

In addition, we examined adult neurogenesis in hippocampi of *App*-KI mice. Cells in the DG positive for DCX, a marker of newborn neurons, were less abundant in *App*-KI mice than in wild-type mice [Fig. 4b, c, t(6) = 3.126, p = 0.0204 by *t*-test]. Cells positive for Ki-67, a proliferation marker, were also less abundant in *App*-KI mice at the same age [Fig. 4b, d, t(6) = 3.07, p = 0.0219 by *t*-test]. Taken together, these data suggest that accumulation of glia and impaired adult neurogenesis were accompanied by Aβ plaque expression in *App*-KI mice at 6 months.

## Discussion

In this study, we used behavioral tests that required proper function of hippocampal and cortical regions [24, 28, 29]. These tasks assess different parameters of cognitive function. The LD task measures pattern separation, the dPAL task assesses object-in-place associative memory, the Morris water maze test assesses spatial reference memory, and the visual discrimination and reversal learning tasks measure cognitive flexibility [8, 23–25]. This is the first study to evaluate cognitive functions in AD model mice using a systematic touchscreen-based behavioral test battery.

We used a touchscreen-based LD task to analyze the pattern separation ability of AD model mice. We utilized an *App*-KI mouse model that exhibits significant Aβ accumulation, increased neuroinflammation, and synaptic alteration in the hippocampus starting at the age of 4 months without overproducing other APP fragments such as the soluble fragment of APP, C-terminal fragment-β, C-terminal fragment-α, or APP intracellular domain [11–13]. *App*-KI mice do not exhibit tau pathology, neurodegeneration, or severe neuron loss, suggesting that they are models of preclinical AD [12]. In these mice, Aβ accumulation in the cortical region starts even earlier (at 2 months) [13]. We also observed a similar pattern of Aβ accumulation (Fig. 4a). In addition, we detected the age-dependent presence of activated microglia and GFAP-reactive astrocytes around Aβ plaques, starting at 4 months, indicating that amyloidosis-associated neuroinflammation occurred at an early stage in *App*-KI mice (Fig. 4a) [30]. Although many researchers have subjected these mice to different classical behavioral tests, behavioral impairments were not reported at the early stage (Table 1) [14–17]. However, using a touchscreen-based LD task in *App*-KI mice, we detected cognitive impairment around 4–5 months of age (Fig. 1). In particular, *App-*KI mice exhibited significant impairment when the stimuli were separated by a large gap (LDl task) (Fig. 1e). By contrast, in the LDs task, we observed no significant difference between the *App-*KI and control groups. It is possible that the gap between stimuli in LDs is important, and it may have been too narrow for the mice to discriminate images. Indeed, in the LDm training, *App-*KI mice exhibited poorer performance than the control group (Fig. 1b and c). At a minimum, these results imply that mild pathological changes had progressed at an early stage in *App*-KI mice. Further experiments are required to prove this hypothesis.

**Table 1:** Summary of significant differences in behavioral tests in *App*-KI mice in this study vs. previous studies

Behavioral tests	Test significance	Significant difference	References
Y-maze	Short-term memory	Yes (6 months)	Saito et al. [13]
Place preference and reversal task	Spatial learning and reversal learning	Yes (13–14 months)	Masuda et al. [16]
Serial reaction time task	Impulsivity and attention	Yes (13–14 months)	
Place avoidance	Extinction learning	Yes (8–9 months)	
Delay discounting task	Compulsive behavior	Yes (8–9 months)	
Novel object recognition	Recognition memory	Not detected (6 months)	Whyte et al. [17]
Y-maze	Working memory	Not detected (6 months)	
Morris water maze	Spatial reference memory	Not detected (6 months)	
Open field	Anxiety	Yes (6 months)	
Fear conditioning	Fear learning	Not detected (15–18 months)	Sakakibara et al. [14]
Elevated plus maze	Anxiety-related behavior	Yes (6–18 months)	
Barnes maze	Spatial memory	Yes (8 months)	
Spatial reversal learning	Flexibility and impulse control	Not detected (8 months)	
Morris water maze	Spatial reference memory	Not detected (10–11 months)	Latif-Hernandez et al. [15]
Cage activity and exploration	Spontaneous activity	Yes (3 and 10 months)	
Social Preference Social Novelty (SPSN) test	Social memory	Not detected (3, 6, and 10 months)	
Location discrimination	Pattern separation	Yes (4–5 months)	Present study
Different object–location paired-associate learning	Paired-associative memory	Yes (4–6 months)	
Visual discrimination, Reversal learning	Cognitive flexibility	Not detected (4–5 months)	
Morris water maze	Spatial reference memory	Not detected (6 months)	

Pattern separation requires the function of complex neuronal networks in the hippocampal subregion (entorhinal cortex–DG–CA3–CA1 circuit) [31–33]. Previous studies showed that these subregions and networks are the primary targets of Aβ pathology during the early stage of AD [34–37]. In *App*-KI mice, accumulation of Aβ plaque is associated with loss of synaptic markers [13]. Moreover, cholinergic synapses are essential for survival, and glutamatergic receptor signaling is required for proper migration and positioning of newborn neurons in DG of adult brain [38, 39], a physiological process that is essential for optimum pattern separation performance in rodents [40–42]. Pattern separation in human is also impaired at the early stage of AD [43, 44] and has been linked to poor hippocampal neurogenesis [43, 45]. In this study, immunohistochemical analysis of both DCX and Ki-67 revealed altered adult hippocampal neurogenesis in 6-month-old *App*-KI mice (Fig. 4b). Notably, in this regard, adult hippocampal neurogenesis is impaired before the onset of classical AD pathology in AD mice [46]. Inflammation-mediated disruption of adult hippocampal neurogenesis impairs pattern separation [47]. Elevated levels of DCX-positive cells enhance performance in hippocampus-dependent tasks [48]. Based on these observations and together with our immunohistochemical data (Fig. 4), we speculate that the cognitive deficit observed in this study was due to Aβ-associated synaptic loss, which altered adult neurogenesis and proper incorporation of newborn cells into the hippocampal network. In fact, synaptic alterations have been reported in the same mouse model: specifically, presynaptic synaptophysin and postsynaptic PSD95 immunoreactivity near the Aβ plaque is reduced [11]. Further research is needed to determine whether the cognitive impairments we observed in the tasks described above are the result of these synaptic alterations.

Next, we assessed the performance of *App-*KI mice in the dPAL task to assess paired-associate memory. Previous studies demonstrated that dPAL, as part of the Cambridge Neuropsychological Test Automated Battery (CANTAB), is very efficient at distinguishing early-stage patients with AD from those suffering cognitive impairment caused by other clinical conditions [49–51]. This task is also sensitive to dorsal hippocampal dysfunction and neurogenesis in DG [22, 29], and both of these factors are affected from the early stage of AD pathology [36, 37, 52]. In accordance with previous studies, our results revealed significant impairment in the *App-*KI group relative to control mice (Fig. 2b), although cognitive function related to object-in-place memory was maintained in the *App*-KI group (Fig. 2c). This result reinforces clinical data showing that the dPAL task provides a sensitive means for detecting AD at its early stage. Our findings are the first data to demonstrate the potential use of touchscreen-based LD and dPAL tasks in detecting cognitive impairments associated with early AD. Moreover, it is possible that our test battery can detect impairment at an earlier age (2–4 months of age) than 4–6 months, because massive accumulation of amyloid β and glial cells were observed in 4–month-old mice (Fig. 4a). We would like to continue the experiments for this point in future studies.

We also assessed the behavioral performance of *App-*KI mice in touchscreen-based visual discrimination and reversal learning, as well as in the classical Morris water maze test (Fig. 3). Neither of these tests revealed any cognitive impairment at the age tested. However, a previous study reported significantly improved performance in TgCRND8 (4.5-months of age) mice during the reversal phase [24]. By contrast, APPSwDl/Nos2^−/−^ (4–5 months of age) and APPS1-21 (6 months of age) mice exhibited impaired performance in the reversal learning task [53, 54]. Medial prefrontal cortex lesions facilitate reversal learning in mice [55], whereas lesions of the orbitofrontal cortex impair reversal learning [56, 57]. It would be intriguing to find out whether different mutations deposit Aβ differentially in specific brain regions. Interestingly, in this study, *App*-KI mice performed similarly to the control group, even though significant Aβ had accumulated in the cortical region at the age tested here. One possible reason for this discrepancy may be the number of trials per session. In this study, the number of trials in a single session was much higher than in the reports described above, which may have compensated for the mild impairment in behavioral flexibility at the early stage of AD. Another possible explanation for this result may be that at the early stage of AD, the hippocampal function is more prone to Aβ-related dysfunction, whereas the cortical regions are more resilient and show impaired function at a much later stage. Therefore, hippocampus-dependent LD and dPAL tasks were impaired but not reversal learning, which depends on the cortical subregion. However, to rule out any involvement of cortical regions in pattern separation or associative learning, further experiments will be required.

In the Morris water maze test, *App-*KI mice exhibited slightly worse performance than the control group, but the difference was not statistically significant. Our result is in accordance with a recent study showing that *App-*KI mice performed similarly to the control group [17]. Executive function related to pattern separation and paired-associate memory is impaired at the early stage of AD, whereas reference memory is impaired at a later stage. Perhaps at the later stage of the disease (11–12 months), the subtle behavioral differences observed in our study may become significant. Moreover, mice usually avoid wet conditions and forced swimming stress may induce a stress tolerance-dependent bias in the outcome of the Morris water maze test. Rodent behavioral tests were historically developed using animals undaunted by wet conditions. In that sense, the touchscreen-based test battery would be superior.

In conclusion, using *App*-KI mice, which recapitulate Aβ pathology without overexpression of APP fragments, we showed that hippocampus-dependent touchscreen-based tasks can detect AD-associated behavioral impairments with high sensitivity at the early stage of the disease when classical tests cannot efficiently assess cognitive impairment. The reason for the sensitive difference between behavioral tasks may be that the required cognitive function and the difficulty of the task differ between these tasks. These data suggest that touchscreen-based tasks could be useful for advancing the translational studies by evaluating the efficacy of candidate therapeutics in rodent models of AD from an early stage.

## Data Availability

The data used in our study are available from the authors on reasonable request.

## Abbreviations

AD: Alzheimer's disease
Aβ: Amyloid beta
APP: Amyloid precursor protein
*App*-KI: *App*^*NL*^^−^^*G*^^−^^*F/NL*^^−^^*G*^^−^^*F*^ Knock-in
CANTAB: Cambridge neuropsychological test automated battery
DCX: Doublecortin
DG: Dentate gyrus
PBS: Phosphate buffer in saline
dPAL: Different paired-associate learning
ITI: Inter-trial interval
LD: Location discrimination
